# Hashimoto’s Thyroiditis Presents as an Acute Encephalopathy: A Case Report of Unusual Presentation

**DOI:** 10.7759/cureus.21130

**Published:** 2022-01-11

**Authors:** Sohaib Khatib, Fouad Jaber, Marwa Elsayed, Islam M Shatla, Majdi S Hamarshi

**Affiliations:** 1 Internal Medicine, University of Missouri Kansas City School of Medicine, Kansas Cty, USA; 2 Internal Medicine, University of Missouri Kansas City School of Medicine, Kansas City, USA; 3 Critical Care Medicine, University of Missouri Kansas City School of Medicine, Kansas City, USA

**Keywords:** altered mental state, autoimmune encephalitis, thyroid peroxidase antibody, encephalopathy, hashimoto's thyroiditis

## Abstract

Hashimoto's thyroiditis is the most common thyroid disorder in the United States. Hashimoto encephalopathy is a rare presentation of Hashimoto's thyroiditis that is frequently misdiagnosed.

We present the case of a 71-year-old female who had normal mental status at baseline. She presented with acute alteration in mental status. Further evaluation with brain MRI showed a hyperintense signal in the bilateral centrum. Spinal fluid analysis revealed elevated protein. Thyroid peroxidase (TPO) antibody was elevated at 59.7 and TSH was elevated at 4.9. Her mental status improved dramatically after treatment with steroids and levothyroxine.

This diagnosis should be suspected when the patient develops acute encephalopathy with positive serum thyroid antibody settings with a complete return to normal mental status after treatment with steroids.

## Introduction

Hashimoto's thyroiditis (HT) is the most common cause of hypothyroidism and also the most common autoimmune thyroid disease [1]. Its annual incidence around the world is about 0.3-1.5 cases per 1000 individuals [2]. It is characterized by autoimmune-mediated destruction of the thyroid, with diffuse lymphocytic infiltration of the thyroid by predominantly thyroid-specific B and T cells and follicular destruction [3]. HT is associated with several complications such as T-cell lymphoma, papillary thyroid carcinoma, and encephalitis.

Acute encephalopathy is a common medical problem secondary to infectious, metabolic, structural, or toxic causes [4]. Here, we report a case of a 71-year-old female who developed acute metabolic encephalopathy secondary to Hashimoto's autoimmune encephalitis.

## Case presentation

The patient is a 71-year-old female with a past medical history significant for hypertension and hyperlipidemia. She was in her usual state of health (patient has normal mental status and works as grant writer) until one week prior to presentation when she had several falls. On presentation, the patient's sister and neighbors reported that the patient had not been herself lately. Her home medications included an angiotensin-converting enzyme (ACE) inhibitor, calcium channel blocker, and beta-blocker medications. Initial physical examination showed the patient was alert, active, and oriented to time, place, and person without focal neurologic deficits. Vital signs were significant for hypothermia, hypotension, and bradycardia, 34 ºC, 78/44 mmHg, 50 beats per minute, respectively. Electrocardiogram (EKG) showed an incomplete left bundle branch block and sinus bradycardia. Further workup was negative including urine drug screen, urine analysis, liver function tests, salicylate level, serum acetaminophen level, and serum alcohol level. TSH level was marginally elevated 4.9 (0.4-4.6), normal free T4 0.9 (0.8-2.6), normal serum cortisol, and ACTH levels. Imaging studies including computed tomography (CT) scan of abdomen-pelvis and CT head were normal.

Beta-blocker and calcium channel blocker overdose and septic shock diagnoses were entertained. The patient was resuscitated with intravenous (IV) fluids, she was also treated with atropine, glucagon, norepinephrine, and epinephrine infusions. The patient was also started on broad-spectrum IV antibiotics and Bair Hugger for concerns about sepsis and hypothermia. High-dose insulin euglycemic therapy was then started for beta-blocker and calcium channel blocker overdose.

**Figure 1:**
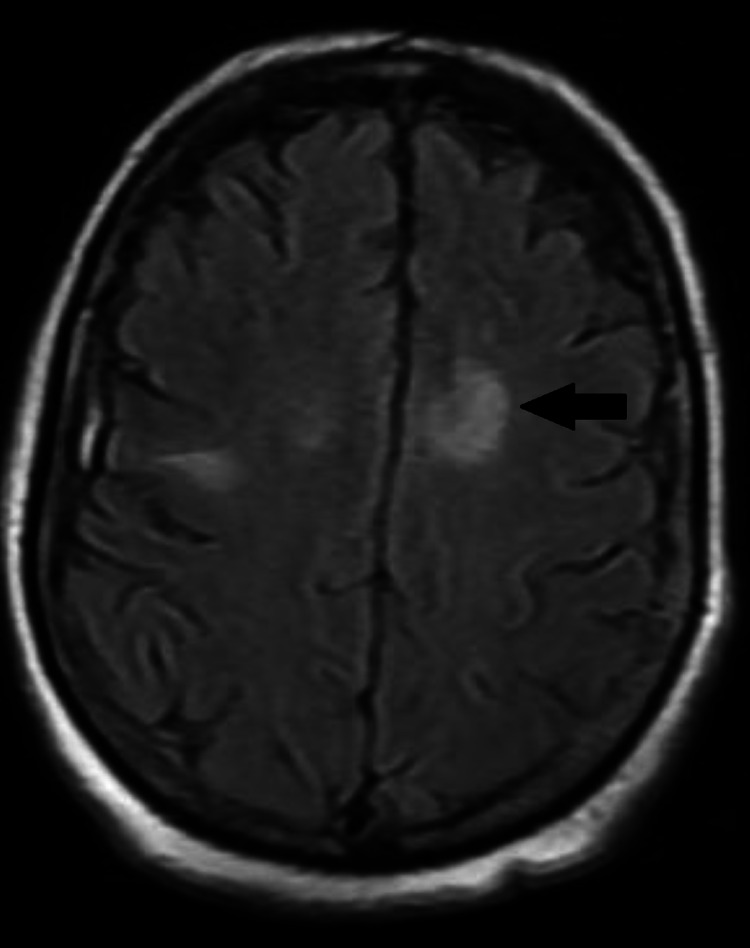
Brain MRI with and without contrast showing confluent T2 hyperintense signals (black arrow) in the bilateral centrum

The next day, the patient developed acute worsening of mental status with a new left-sided facial drop. Physical examination showed bilateral upper extremities drift, lower extremities weakness, aphasia, and dysarthria. After this acute change in mental status and neurological exam, CT head and neck angiogram was done and revealed no acute intracranial process with no aneurysm or arterial stenosis. MRI brain with-without contrast was negative for acute infarct. It showed chronic infarct in the left parieto-occipital cortex. It also showed a confluent T2 hyperintense signal in the bilateral centrum (Figure 1).

The patient’s encephalopathy continued to worsen with persistent dysarthria, visual hallucinations, and eye deviation. The patient was then intubated and placed on mechanical ventilation for concerns of airway protection. Electroencephalogram (EEG) showed findings consistent with mild multifocal subcortical dysfunction. Lumbar puncture (LP) with cerebrospinal fluid (CSF) analysis showed a significant finding of elevated protein > 600 mg/dL (Table 1).

**Table 1:** Cerebrospinal fluid (CSF) analysis with significantly elevated CSF protein finding

CSF test	Result	Normal result
CSF color	Colorless	Colorless
CSF Xanthochromia	Negative	Negative
CSF WBC count	6	0-5
CSF RBC count	2	0
CSF neutrophils	21%	0%-6%
CSF lymphocytes	37%	40%-80%
CSF glucose	134	40-70 mg/dL
CSF protein	>600	15-60 mg/dL
CSF Angiotensin Converting Enzyme	0.4	0.0-2.5 U/L
CSF West Nile virus IgG/IgM	Negative	Negative
CSF autoimmune encephalitis panel	Negative	Negative

Autoimmune encephalitis was suspected; however, the CSF autoimmune encephalitis panel (this included Anti-Neuronal Nuc Ab, Tp 1, Anti-Neuronal Nuc Ab, Tp 2, Anti-Neuronal Nuc Ab, Tp 3, Anti-Glial Nuclear Ab, Type 1, Purkinje Cell Cyto Ab, Tp 1, Purkinje Cell Cyto Ab, Tp 2, Purkinje Cell Cyto Ab, Tp Tr, Amphiphysin Ab, CRMP-5-IgG, Striated Muscle Aby, P/Q-Type Calcium Channel Aby, N-Type Calcium Channel Aby, AChR Ganglionic Neuronal Aby, and VGKC-Aby) was negative. Further workup for acute encephalopathy showed a positive anti-nuclear antibody (ANA) panel with a titer of 1:160, negative SS-A/Ro antibodies, SS-B/La antibodies, and anti-Smith antibodies. In addition, vitamins and minerals with B1, B6, B12 vitamins, copper, folate, and zinc levels were within the normal limits.

Further workup with thyroid peroxidase (TPO) antibody showed elevated levels at 59.7 IU/mL, this raised concerns about Hashimoto's autoimmune encephalitis. The patient was then started on IV methyl-prednisone 500 mg twice daily for five days; she was also started on oral levothyroxine 75 mcg daily. The patient had significant improvement in her mental status after that, and the patient was entirely back to her normal baseline mental status on day 5 of steroids. The patient was then extubated and discharged on oral prednisone 60 mg with a slow taper over several weeks in addition to oral levothyroxine 75 mcg daily.

## Discussion

Hashimoto encephalopathy (HE) is one of the less common presentations of HT [4]; however, the disease might be underrecognized, and the prevalence may be as high as 0.2% [4]. Autoimmune pathogenesis [5] of HE seems to be the most acceptable one, and it is assumed based on the presence of anti-thyroid antibodies (ATA) and response to steroids or immunotherapy.

The exact mechanism that promotes the formation of ATA is not well established. Nonetheless, these pathologic antibodies may develop as cross-reacting to a foreign antigen [6]. Also, no direct link has been established yet between the degree of elevation of ATA and the severity of HE. It is suggested that ATA are the hallmark of HE rather than playing a direct role in the pathogenesis of HE [7]. Hence, the more accurate name for this condition is steroid-responsive encephalopathy associated with autoimmune thyroiditis (SREAT) [8,9].

There is a broad spectrum of presenting manifestations; however, encephalopathy manifesting with abrupt confusion with an altered level of consciousness is the hallmark presentation [8,10]. Two patterns of cognitive dysfunction are predominant; a stroke-like pattern which is more acute to subacute, and a gradual diffuse progressive pattern which might result in dementia, hallucination, or even lethargy, progressing to coma in extreme cases [8,10].

Accompanying non-cognitive symptoms include seizure (in approximately two-thirds of patients), myoclonus (seen in up to 38%), loss of consciousness [10], tremors, hyperreflexia, poor appetite, ataxia, and dysarthria [8]. Besides, psychosis, in the form of paranoid delusion and visual hallucinations, has been reported in 25%-36% [4,8]. Anxiety, depression, hypersomnia, and difficulty initiating sleep are initial presenting symptoms [11]. Depression, anxiety, emotional lability, and social withdrawal have been reported as sole symptoms [12].

In our case, the patient presented with recurrent falls and observed changes in her mental status and thought process prior to admission. She then developed a stroke-like pattern and visual hallucinations during hospitalization, which are common manifestations in HE. A concurrent diagnosis of beta-blocker overdose and calcium channel blocker overdose is possible, but worsening acute encephalopathy despite resuscitation measures was not fully explained by the overdose.

HE is a diagnosis of exclusion; toxins-related, infectious, metabolic, neoplastic, and autoimmune causes should be excluded [3]. It is suspected based on highly variable neurological and neuropsychiatric manifestations accompanied by normal or nonspecific MRI findings, elevated serum ATA concentration, and clinical response to corticosteroid therapy. Neuroimaging studies, EEG, and CSF analysis can be supportive but not diagnostic. A detectable level of ATA must be present to consider HE [12]. Analysis of CSF should be performed; the most common findings are elevated proteins and less commonly pleocytosis [13]. Thyroid hormones should be measured; however, normal values do not exclude HE diagnosis [10]. EEG may detect generalized slowing related to encephalopathy or the presence of subclinical seizure activity. MRI brain is usually perfumed to exclude structural causes of encephalopathy [11]. Over half of patients with HE will have a normal brain and spine MRI; however, inflammatory lesions and nonspecific abnormalities may be observed or develop over time, and cerebral atrophy may occur later [14]. CT scan of the brain is usually normal but may show cerebral atrophy or ventricular dilatation [15].

Several diagnostic criteria have been developed. Diagnostic criteria were put by Castillo et al. [9], as outlined in Table 2. In our report, given the fact that there was no previous psychiatric history, presenting encephalopathy, the rapid onset of the psychotic features, subclinical hypothyroidism, no other probable causes of encephalopathy in addition to the presence of anti-thyroid peroxidase antibody (TPOAb), high CSF proteins, and response to steroids made the diagnosis of HE as the most likely diagnosis.

**Table 2:** Diagnostic criteria for Hashimoto’s encephalopathy TSH - thyroid-stimulating hormone; CSF - cerebrospinal fluid

Criteria
- Encephalopathy manifested by cognitive impairment and one or more of the following: neuropsychiatric features (hallucinations, delusions, or paranoia), myoclonus, generalized tonic-clonic or partial seizures, or focal neurologic deficits
- Presence of serum thyroid antibody
- Euthyroid status (TSH: 0.3-5.0 mIU/L) or mild hypothyroidism (TSH: 5.1-20.0 mIU/L) that would not account for encephalopathy
- No evidence in blood, urine, or CSF analyses of an infectious, toxic, metabolic, or neoplastic process
- No serologic evidence of autoantibodies to indicate another diagnosis
- No findings on neuroimaging studies indicating vascular, neoplastic, or other structural lesions to explain the encephalopathy
- Complete or near-complete return to the patient's neurologic baseline status with steroid treatment

The mainstay therapy for HE is usually corticosteroids, in addition to the treatment of any concurrent hypothyroidism. Administration of steroids is crucial to establish steroid responsiveness by nonspecifically targeting the central nervous system (CNS) inflammation with a response rate as high as 90%-98% [9]. Currently, no guidelines define the treatment duration, optimal dose, and rate of tapering of corticosteroids, and it is usually guided by clinical response [9]. The optimal dose has yet to be established, but the most used regimen is an initial high dose (500-1,000 mg of methylprednisolone per day for adults or 30 mg/kg of body mass for children) administered for 3-7 days, followed by prednisone (1-2 mg/kg/days, maximum 60 mg/days) for six to eight weeks. Tapering of dose should be performed gradually. Initially, prednisone doses ranging from 50 to 150 mg daily have also been used with no significant benefit of one treatment versus another [16]. Other immunosuppressive medications such as azathioprine, cyclophosphamide, methotrexate, and mycophenolate mofetil are an option in cases of corticosteroids intolerance, failure to respond, or relapse [17]. IV immune globulin (IVIG) [18] or plasmapheresis may also be used [19].

We treated our patient with 500 mg IV methyl-prednisone twice daily and oral levothyroxine 75 mcg daily for five days. The patient had a complete return to baseline on day 5 of steroids followed by six-eight weeks' taper using oral prednisone.

The prognosis of SREAT is generally good, and symptoms typically resolve or improve within a few months.

## Conclusions

Acute encephalopathy has a broad differential diagnosis and can be multifactorial. Hashimoto's autoimmune encephalitis is an unusual cause of acute alteration in mental status. Physicians should consider it in the differential diagnosis of acute encephalopathy. It is deemed to be a diagnosis of exclusion and the diagnosis is usually suggested with elevated protein in CSF analysis and positive ATA in serum. This condition can be effectively treated with corticosteroids in addition to the treatment of concurrent hypothyroidism.
